# Role of Apoptosis in HIV Pathogenesis

**DOI:** 10.1155/2022/8148119

**Published:** 2022-04-14

**Authors:** Cyril Jabea Ekabe, Njinju Asaba Clinton, Eugene Kusi Agyei, Jules Kehbila

**Affiliations:** ^1^Grace Community Health and Development Association, P. O. Box 15, Kumba, Southwest Region, Cameroon; ^2^Mbonge District Hospital, Mbonge, South West Region, Cameroon; ^3^Faculty of Health Sciences, University of Buea, Buea, Cameroon; ^4^Health and Empowerment Foundation, Kumba, Cameroon; ^5^Faculty of Pharmacy and Pharmaceutical Sciences, Kwame Nkrumah University of Science and Technology, Kumasi, Ghana; ^6^Wum District Hospital, Wum, Northwest Region, Cameroon

## Abstract

The apoptotic pathway is an important cell death pathway that contributes to the maintenance of homeostasis in living systems. However, variations in apoptosis have been linked to many diseases such as cancers and chronic infections. The HIV infection has contributed to increase mortality and morbidity worldwide, predominantly through the induction of gradual depletion of CD4+ T cells. The induction and mediation of both the intrinsic and extrinsic apoptotic pathways are crucial in HIV pathogenesis and intracellular survival. Consequently, a deep molecular understanding of how apoptosis is induced and modulated in HIV-mediated CD4+ T cell depletion is paramount, as this can lead to new portals of therapeutic intervention and control.

## 1. Introduction

The human immunodeficiency virus (HIV) is a single stranded positive sense retrovirus belonging to the lentiviruses that primarily infect CD4+ T cells, macrophages, and dendritic cells. Its genome consists of three open reading frames containing the gag, pol, and env genes, which encode viral core proteins, reversed transcriptase/integrase, and viral envelop glycoproteins, respectively. The virus contains regulatory and accessory genes that encode regulatory proteins (Tat and Rev), and accessory proteins (Vif, Vpr, Vpu, and Nef), respectively. The genome is flanked by long terminal repeats that are pivotal for viral transcription, reversed transcription, and virus integration [1]. The HIV virus infects CD4+ T cells, macrophages, and dendritic cells by binding to the CD4+ receptors via the viral envelop protein gp120, after which viral entry is mediated by the interaction between virus gp41 and coreceptors(CXCR4 in CD4+ T cells, or CCR5 in monocytes/macrophages, dendritic cells, and activated T cells) [1].

The pathogenicity of HIV infection results from the gradual chronic depletion or loss of CD4+ T cells in untreated patients, predisposing patients to opportunistic infections, chronic inflammation, and malignancies characterizing the disease AIDS [2]. The underlying mechanisms contributing to the loss of CD4+ T cells generally can be due to increased destruction, decreased production, or redistribution of CD4+ T cells. Studies have demonstrated that thymic T cell production decreases in HIV-infected individuals compared to uninfected individuals. This was attributed to the direct cytopathic effect of the virus on HIV-infected thymocyte precursors and apoptosis of uninfected thymocyte precursors. Nevertheless, TREC studies were able to confirm the return of thymic function in children after antiretroviral treatment [2]. These studies confirmed that HIV infection has a reversible negative effect on thymic function and size. Considering the effect of HIV on the CD4+ T cells, further investigation was carried out to confirm the relationship between viral load and apoptosis. Conversely, it was confirmed that there was no correlation between apoptosis of circulating CD4+ T cells and viral load [3]. To untangle this dilemma, a study demonstrated a greater degree of apoptosis in bystander non-infected CD4+ T cells of the lymph node than in infected cells. Likewise, other studies have confirmed greater degree of death in non-infected CD4+ T-cells than in infected HIV CD4+ T cells [4]. Hence, attesting to the fact that apoptosis occurs more in non-permissive CD4+ T cells.

Apoptosis is a regulated form of cell death in which the cell is programmed to die through coordinated activation and execution of multiple controlled steps [5]. It is characterized by cell shrinkage, chromatin condensation, nuclear fragmentation, and plasma blebbing. The apoptotic pathway is divided into the extrinsic and intrinsic pathway. The extrinsic pathway is initiated by activation of death receptors on the cell surface belonging to the tumor necrosis factor receptor subfamily. It consists of five well-characterized death receptors, namely, Fas/Apo/CD95 (TNFRSF6), DR3 (TNFRSF25), DR4(TNFRSF10A), DR5 (TNFRSF10B), and DR6 (TNFRSF21) [6, 7]. The binding of the appropriate ligand to the death receptors triggers the extrinsic apoptotic pathway via the formation of the death inducing a signaling complex (DISC). The formation of DISC leads to the activation of caspase 8 that further activates the executioner caspases such as caspase 3 and 7 leading to apoptosis [7]. The major extrinsic apoptotic pathways important in HIV infection include the Fas/FaL, TNF-related apoptosis-inducing ligand (TRAIL), and tumor necrosis factor (TNF) alpha pathways. On the other hand, the intrinsic pathway is tightly regulated by the Bcl-2 gene family of proteins that controls the release of specific factors and caspases from the mitochondria. The net effect of this pathway is the release of cytochrome-c from the mitochondria that forms a complex with APAF1. This complex triggers the activation of caspase 9 which in turn activates the executioner caspases that are responsible for apoptosis induction [8]. The apoptotic pathway plays an important role in development, physiological processes, and homeostasis [9, 10]. With regards to the immune system, the role of apoptosis in T cell development has been elaborated well [11, 12]. Also, apoptosis plays a role in the elimination of dysfunctional cells, tumor cells, and pathogens [13]. Distortion of apoptosis has been associated with cancer, autoimmune diseases, and spread of viral infections such as HIV [13]. The HIV infection is characterized by the presence of viral proteins that have both proapoptotic and antiapoptotic qualities. This includes the gp120, Tat, Vpr, Vpu, and Nef proteins [14]. In this review, we will elucidate the role of the apoptotic pathway in HIV infection and how this pathway can be exploited in developing new avenues of HIV therapy.

## 2. Mechanism of Apoptosis in HIV-Infected and Non-Infected CD4+ T Cells

It has been well demonstrated that HIV induces more cell death in non-infected CD4+ T cells than in infected CD4+ T cells [4]. In addition, a greater proportion of bystander death of non-permissive or resting CD4+ T cells occurs in the lymph nodes rather than in the blood stream. Generally, retroviruses fuse with target cells either as cell-free virions or through cell-to-cell spread. The cell-to-cell spread of viruses occur more in the lymph nodes than in the blood stream, thereby increasing the infectivity of the virus [15]. This is an important point as cell-to-cell spread increases the propensity of contact between infected and naïve/non-permissive cells, predisposing them to bystander cell death. Furthermore, this bystander killing of uninfected CD4+ T cells in the lymph nodes is crucial in the depletion of CD4+ T cell and progression of HIV infection. However, how does the depletion of uninfected cells affect the HIV survival, given the fact that most retroviruses usually infect cells without killing their host? Most probably, the bystander killing of uninfected CD4+ T cells is advantageous to the virus. This is because invasion of these cells mostly leads to abortive infections, thus blocking HIV replication and spread. Various mechanisms have been exploited by the virus to achieve this task, this includes the TNF-a, TRAIL, and Fas/FasL pathways via various viral factors or proteins (for example, gp120 Tat, Vpu, and Vpr). These proteins are generally secreted via HIV exosomes [16] or directly across the cell membrane as soluble proteins. The secretion and binding of these viral proteins to uninfected cells triggers the activation of various apoptotic pathways leading to cell death. The apoptotic pathways induced during HIV infection are described below.

## 3. TRAIL Apoptotic Pathway

The TRAIL is a member of the TNF subfamily that binds to four membrane receptors and one soluble receptor. These receptors exist as type II transmembrane proteins [17], which are cleaved by cysteine proteases [18] through a process called shedding [18] to release soluble forms of TRAIL.

The TRAIL-TRAIL receptor system composes of proapoptotic receptors (TRAIL-R1 (DR4) and TRAIL-R2 (DR5)) and alternative receptors; TRAIL-Rs (TRAIL-3R (DCR1) and TRAIL-4(DCR2), osteoprotegrin) act as soluble decoy receptors and negatively regulate apoptosis induction by TRAIL [19, 20]. This is probably due to the absence of a cytoplasmic tail or truncated tail in TRAIL-R3 and TRAIL-R4, respectively. However, more investigations are required to confirm whether indeed these functions are exerted by these so-called decoy TRAIL receptors in cancer cells [21–23]. The proapoptotic TRAILs exist as dimers in unbound states and form ligand-induced trimers upon binding to their ligands. The TRAIL-R1 and TRAIL-R2 can form either homotrimers or heterotrimers via multimerization or crosslinking of neighboring trimers [24]. Structurally, the proapoptotic TRAIL receptors differ from each other. The main difference is due to the presence of one spliced variant in TRAIL-R1 compared to the two spliced variants in TRAIL-R2 [19]. Likewise, TRAIL-R1 effectively induces apoptosis in cancerous cells than TRAIL-2R [25, 26]. This is due to the higher apoptotic threshold observed in TRAIL-R2 compared to TRAIL-R1 [27].

The proapoptotic TRAIL pathway is divided into the canonical and the noncanonical pathway. The canonical pathway begins with the binding of TRAIL to TRAIL-R1, or TRAIL-R2. Upon binding of TRAIL to its cognate receptor, the intracellular death domains (DDs) of the ligand induce a conformational change that recruits the adaptor molecule FAS-associated death domain protein (FADD) vis DD-DD protein interaction [19]. The adaptor molecule then binds to the initiator caspases 8/10 via DED-DED protein interaction leading to the formation of the TRAIL-DISC complex. The binding and activation of caspase 8 is potentiated by cullin-3-mediated ubiquitination caspase 8 that facilitates p62 binding and assembly of caspase 8 at the DISC [28]. The activation of caspase 8/10 leads to subsequent activation of caspase 3, which initiates the process of apoptosis. This process is regulated by a FLICE-like inhibitory protein (FLIP), which is a caspase 8 homolog that competitively binds to FADD and blocks the apoptotic pathway [29]. Additionally, de-ubiquitination and proteasome degradation of caspase 8 contributes to the regulation of apoptosis [30]. The noncanonical pathway involves the binding of TRAIL to TRAIL-R1 and TRAIL-R2 and subsequent activation of NF-*κ*B via receptor-interacting serine/threonine protein kinase 1 (RIPK1), which triggers phosphorylation of I*κ*B and release and nuclear translocation of NF-*κ*B (23).

## 4. Tumor Necrosis Factor-*α* (TNF) Apoptotic Pathway

Tumor necrosis factor-*α* (TNF) is a proinflammatory cytokine produced by many immune cells such as monocytes/macrophages. This cytokine binds as a trimer to two TNF receptors (TNFR1 and TNFR2) during which it exerts most of its functions [31]. The TNF receptors are composed of a conserved extracellular domain made up of cysteine rich motifs, and a unique intracellular domain made of different motifs that defines the function of a specific TNF receptor [32, 33].

The TNFR1 differs from TNFR2 in the fact that it contains a death domain that has a cytoplasmic region capable of triggering proapoptotic death in cells [34]. The TNF cytokine is produced by immune cells in response to pathogen-associated molecular patterns (PAMPS) and danger-associated molecular patterns (DAMPs). In response to the abovementioned signals, a membrane protein called the TNF-*α* converting enzyme (TACE) cleaves the TNF precursor bound to plasma proteins into soluble TNF forms [35]. In addition, it is worth noting that the plasma membrane-bound TNF and soluble TNF bind to TNFR2 and TNFR1, respectively [36]. Upon binding of TNF-*α* to an appropriate receptor, many signaling pathways are triggered. This includes NF-*κ*B, p38 mitogen-activated protein kinases (MAPK), extracellular signal-regulated kinase (ERK), and c-Jun N-terminal kinase (JNK) pathways. However, we will restrict our discussion only to the apoptotic pathway. The binding of TNF-*α* to TNFR1 death domain triggers a protein-protein interaction between the cytoplasmic tail of TNFR1 and the adaptor protein TNFR-associated death domain (TRADD). This further leads to the recruitment of TRADD which interacts with the dead domain of FADD and subsequently activates the apoptosis pathway via the activation of the caspase 8/10 and caspase 3 [36].

## 5. Fas/FasL Apoptosis Pathway

An attractive insight into the mechanism used by many viruses including the HIV to escape from immune clearance and foster disease progression has been attributed to the widespread virus-induced programmed cell death (apoptosis) including the Fas-Fas Ligand (Fas-FasL) pathway [37]. The Fas receptor (CD95) is a type I membrane protein belonging to the tumor necrosis factor (TNF) receptor family [38]. It has both extracellular and intracellular domains, which compose of three cysteine-rich repeats and a death domain, respectively [39]. The Fas protein is a ubiquitous protein in the body stored within the cytoplasm of cells. It can traffic to the cell surface to form a membrane-bound receptor protein, which is widely expressed in normal and diseased tissues [40]. Its ligand (FasL or CD95L) is a type II membrane-bound protein that belongs to the TNF superfamily [41]. The FasL is also stored in the cytoplasm in secretory vesicles. It is mainly expressed on the cell surface of immune cells such as lymphocytes and natural killer (NK) cells as a 40-kDa membrane-bound protein (mFasL) [42]. The 40-kDa membrane bound form is released from the cell surface through cleavage induced by matrix metalloproteinases to form a 28-kDa soluble protein (sFasL) [43]. Some reports have shown that this sFasL is less efficient at inducing apoptosis in many cell types compared to the mFasL [44] but can act as a chemotactic factor for neutrophils [45]. The Fas-FasL pathway plays a significant role in the maintenance of immune homeostasis. This pathway is pivotal for the destruction of virus-infected cells or cancer cells by cytotoxic T cells and NK cells [42]. The binding of the FasL to its cognate Fas receptor triggers a cascade of tightly regulated downstream signaling events that leads to apoptosis [38]. The Fas protein trimerizes upon binding to FasL and through its cytoplasmic death domain (DD), it interacts with the signaling adaptor molecule FADD (Fas-associated death domain). FADD contains a death effector domain (DED), which by a homologous interaction, recruits the DED-containing zymogenic form (inactive form) of caspase-8 protein to generate the death-inducing signaling complex (DISC) [46, 47]. Upon recruitment, the inactive caspase-8 oligomerizes and becomes activated through self-cleavage [48]. Activated Caspase-8 then cleaves and activates other downstream effector caspases including caspase-3 [47]. It has been reported that activated caspase-8 can either activate procaspase-3 by direct cleavage or through a series of complex interactions involving the Bcl2 interacting protein (BID) and the mitochondria [49, 50].

## 6. Mitochondria Pathway of Apoptosis

**Figure 1:**
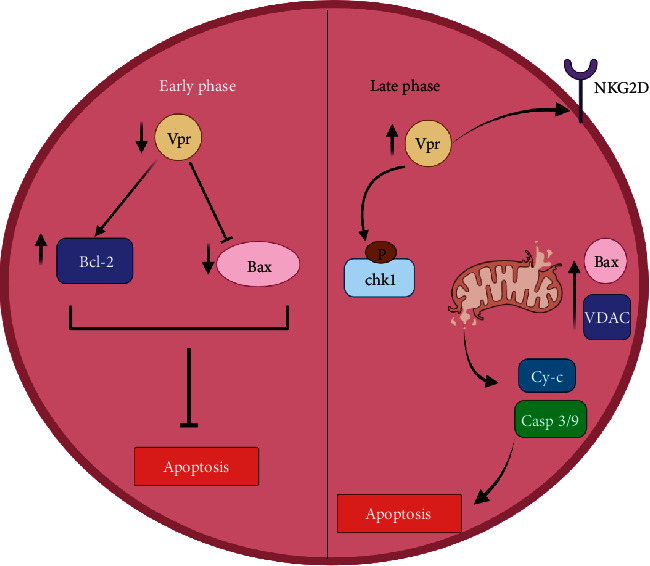
Antiapoptotic and proapoptotic effects of Vpr in the early and later stages of HIV replication.

A diverse array of nonreceptor-mediated stimuli constitute the signaling factors that initiate the mitochondria pathway of apoptosis. The deprivation of certain growth factors, cytokines or hormones, that would have otherwise suppressed death programs constitute such negative stimuli which lead to triggering of apoptosis. On the contrary, positive stimuli includes toxin, hypoxia, hyperthermia, free radicals, and viral infections. [51]. These stimuli induce changes in the inner mitochondrial membrane that leads to the opening of the mitochondrial permeability pore (MPT), loss of the mitochondrial transmembrane potential, and ultimately resulting in the release of two main group of proapoptotic proteins that are usually sequestered in the intermembrane space of the mitochondria into the cytosol [52]. The first group of these proapoptotic proteins include cytochrome-c, Smac (Second mitochondria-derived activator of caspase)/DIABLO (direct IAP binding protein with low pI), and the serine protease HtrA2/Omi [53–55]. These proteins activate the caspase-dependent mitochondrial pathway. Cytochrome-c for instance forms an apoptosome by binding and activating Apaf-1(Apoptotic protease activating factor 1) as well as procaspase 9 [56, 57]. It is the clustering of procaspase 9 in this form that results in the activation of caspase-9, Smac/DIABLO, and the serine protease HtrA2/Omi. The released cytochrome-c triggers the activation of caspase-3 through the formation of the cytoC/Apaf-1/caspase-9-containing apoptosome complex [58]. The released SMAC promotes the activation of caspases by neutralizing the inhibitory effects of inhibitor of apoptosis proteins (IAPs) (see Figure 1) [59]. The IAPs encompass a group of endogenous inhibitors of caspases harboring one or more of the baculovirus IAP repeat (BIR) domains that mediate their inhibitory interaction with caspases [59]. Once activated, caspase-3 induces the breakdown of several cytoskeletal/structural proteins and other nuclear signaling proteins Alternatively, it has also been reported that caspase-3 can cleave the inhibitor of caspase-activated DNAse (ICAD), which frees CAD to enter the nucleus and cleaves DNA, thereby inducing apoptosis [51, 60]. This pathway is highly regulated by members of the Bcl-2 family of proteins [61]. Each member of this family is characterized by one or more of the Bcl-2 homology (BH) domains (BH1-BH4). Their special significance is underscored by the fact that they can determine if the cell commits to apoptosis or halts the process. This family of protein controls the mitochondrial membrane permeability and can be either proapoptotic (BCL-10, BAX, BAK, BID, BAD, BIM, BIK, and BLK) or antiapoptotic(BCL-2, BCL-X, BCL-XL, BCL-XS, BCL-W, and BAG).

Apoptosis is modulated by HIV proteins.

### 6.1. Nef Proteins and Apoptosis

**Figure 2:**
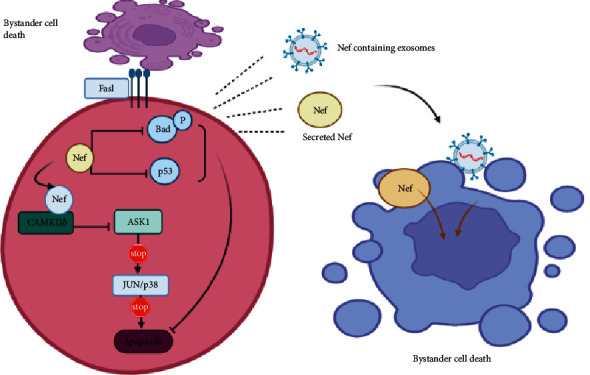
Role of Nef proteins in inducing bystander cell death.

Nef protein is a conserved HIV accessory protein measuring 27–34 kDa and is highly expressed during the early stages of HIV viral replication cycle [62]. This protein triggers the decrease in expression of CD4 and MHC-1 and modulates many signaling pathways [63]. Furthermore, the Nef proteins have been shown to portray both proapoptotic and antiapoptotic characteristics. This depends on different factors, including whether the cell is an infected or uninfected cell. In infected cells, Nef protein induce different mechanisms which blocks cell death and prolongs cell survival for viral replication. It has been shown that Nef proteins bind to the TCR-*ζ* chain and increase the expression of FasL during early HIV infection [64]. The binding of Nef to TCR-*ζ* chain requires co-stimulation for activation of T cell signaling required for HIV replication. Despite the expression of FasL induced by Nef, these cells escape apoptosis in early infection via the downstream effect of Nef on key molecules. The Nef protein binds and blocks the downstream activity of apoptosis signaling regulatory Kinase 1(ASK1) [64]. The ASK1 protein is important in the downstream activation of Fas and TNF alpha death pathways, hence blocking of the activity of ASK1 by Nef increase the survival of infected cells. The underlying mechanism involves the binding of CAMKII*δ* to the C terminal of Nef to form a CAMKII*δ*-Nef complex. This complex binds to ASK1, hindering the activation of the JUN/p38 pathway required for apoptosis [65]. Second, Nef proteins inhibit the intrinsic pathway of apoptosis by binding to PI-3 kinase and mediating the activation of Nef-associated PAK kinase. This leads to phosphorylation and inactivation of the proapoptotic factor called Bad, thereby leading to the inhibition of apoptosis [64]. Third, it has also been shown that Nef inhibits apoptosis in infected cells by activating p53 ubiquitination hindering the activation of apoptosis and favoring cell survival. The underlying mechanism involves the binding of Nef to E6AP promoting E6AP-dependent p53 ubiquitination and subsequent proteasomal degradation [66]. Nevertheless, Nef proteins display proapoptotic characteristics. This has been proficient especially in uninfected cells (Figure 2). The molecular mechanisms involve the activation of the Fas-FasL pathway via the upregulation of FasL in infected cells [64]. Furthermore, HIV Nef proteins secreted in exosomes from infected cells have been shown to trigger apoptosis in bystander CD4+ T cells [62]. The Nef-containing exosomes enter resting cells by endocytosis and probably trigger inappropriate activation of TCR in resting cells leading to bystander cell death. In appropriate activation of TCR in uninfected cells could lead to impaired secretion of factors such as NF*κ*b, NFAT, AP-1, and AP2. The interaction of AP1 and AP2 with Nef proteins has been elucidated to block the activation of Nef-mediated apoptosis [67]. Thus, the Nef-mediated impairment of AP1 and AP2 production in uninfected cells contributes to increased bystander CD4+ T cell apoptosis.

### 6.2. Tat Proteins and Apoptosis

**Figure 3:**
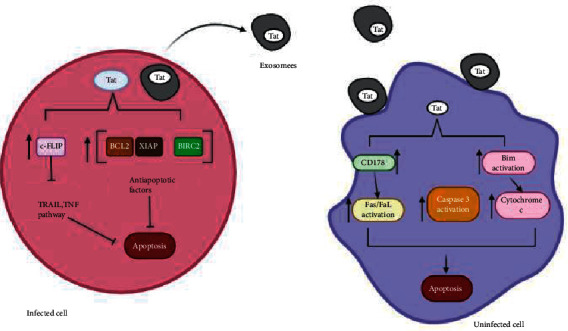
Role of Tat exosomes and secreted Tat in the induction of apoptosis in uninfected cells.

The Tat protein encoded by the regulatory Tat gene plays a paramount role in HIV transcription. This protein acts as a transcriptional factor by binding to TAR (Trans-activator Response Element) from the HIV RNA molecule, enhancing HIV transcription by preventing premature termination of transcription [68]. Tat proteins play a crucial role in inducing apoptosis, especially in bystander cells via different mechanism. It has been shown that Tat proteins secreted in exosomes or directly from infected HIV CD4+ cells are internalized via caveolae mediated internalization by many cell types [69]. Following internalization, Tat proteins induce apoptosis in bystander cells by activation of both the intrinsic and extrinsic apoptotic pathways (Figure 3). The Tat protein activates the proapoptotic factor Bim in the mitochondria that triggers activation of the intrinsic apoptotic pathway leading to death of uninfected cells [69]. Furthermore, Tat proteins increase the expression of CD178 in infected cells leading to the activation of the Fas/FasL extrinsic apoptotic pathway [70]. Again, Tat proteins triggers activation of caspase 3 and poly A-(A/DP)-ribose polymerase to augment apoptosis [68]. Tat proteins further inhibit apoptosis in infected cells by upregulating c-FLIP (an inhibitor of FADD, and Caspase 8), thereby inhibiting the TRAIL, and TNF extrinsic apoptotic pathways [71]. Also, especially in infected cells, Tat upregulates various antiapoptotic factors (BCL2, X-linked inhibitor of apoptosis (XIAP), and Baculoviral IAP repeat containing 2(BIRC2)) essential for increasing cell survival for viral replication [72].

### 6.3. Vpr protein and apoptosis

The Vpr protein is one of the accessory proteins expressed during the late phases of HIV transcription. This protein portrays both antiapoptotic and proapoptotic characteristics depending on the period of the viral replication cycle in infected cells (Figure 1). At the early phases of the viral cycle, low concentration of Vpr inhibits apoptosis by activating and upregulating Bcl-2 and downregulating proapoptotic factors such as Bax, thus blocking the activation of the intrinsic apoptotic pathway [73]. On the contrary, Vpr concentration increases at the late level of HIV viral replication cycle. The increased level of Vpr has been linked to arrest of the G2 phase of the cell cycle and activation of apoptosis [74]. The molecular mechanism involves activation of Bax, porin complex, and voltage-dependent anion channels (VDACs) leading to the disruption of the mitochondria membrane and release of cytochrome c and activation of the intrinsic apoptotic pathway [75]. This is probably due to prolonged Vpr induced cell arrest which disfavors Viral replication and survival. Furthermore, Vpr mediated phosphorylation of CHK1 during the S phase of the cell cycle also induces apoptosis [73]. Other mechanisms induced by Vpr such as upregulation of NKG2D stress ligands in CD4+ T cells trigger natural killer cell-mediated apoptosis [76]. Moreover, Vpr can also induce apoptosis in uninfected cells via the same mechanisms because it is secreted like soluble proteins into the extracellular space [73].

### 6.4. Virus protein U(Vpu) protein and apoptosis

The Vpu protein is one of the accessory HIV proteins that downregulates CD4 via proteasomal degradation [77] and regulates the release of viral particles via inactivation of BST-2 [77]. This protein induces apoptosis in infected cells at the late stages of infection. This probably occurs after sufficient HIV replication, favoring the release of virions that will infect other cells. The molecular mechanism underlying activation of apoptosis in infected individuals has been described. It has been well illustrated that Vpu induces the deregulation of I*κ*B, thereby inhibiting NF*κ*B synthesis which is necessary for the activation of antiapoptotic factors such as the Bcl-2 family [78]. Furthermore, HIV Vpu blocks *β*-TrcP–dependent ubiquitination of p53, thereby preventing proteasome degradation of p53, thereby favoring activation of apoptosis [79].

### 6.5. Glycoprotein (Gp)120 and apoptosis

**Figure 4:**
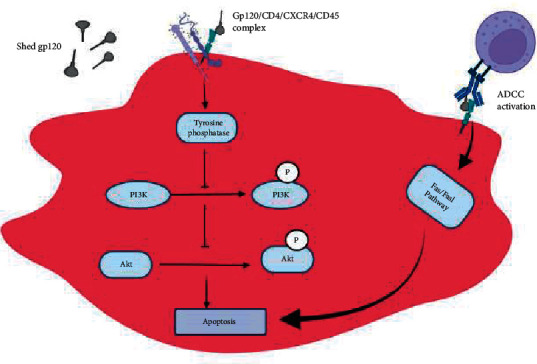
Role of shed gp120 in inducing inactivation of P13K/Akt pathway following the engagement of CD4+5 after gp120/CD4+ interaction. Also, as illustrated, gp120 forms a complex with CD4+, which is eventually recognized by antibodies that trigger ADCC activation.

The Gp120 protein is a major envelop glycoprotein important in HIV pathogenesis. Interestingly, increased levels of gp120 is reported in the blood and tissues of HIV patients [80]. Serum gp120 has the capability of binding to uninfected T cells via its interaction with CD4 receptor. This binding contributes remarkably to bystander destruction of T cells via different mechanisms (Figure 4) [81]. The binding of gp120 to CD4 receptors in uninfected cells contributes markedly to the regulation of Akt function. Akt in its phosphorylated state increases cell survival and blocks cell death. However, it has been well illustrated that the binding of gp120 to uninfected Jurkat T cells and peripheral blood mononuclear cells decreases the phosphorylation of Akt leading to apoptosis [82]. The ability of gp120 to induce Akt phosphorylation is linked to CD45 phosphatase, an important molecule necessary for T cell activation. There has been previous evidence associating CD45 and cell death of thymocytes and PBMCs [83]. Also, the gp120/CD4 interaction triggers confirmational changes in the CD45 molecule that induces interaction between CD45 and the chemokine receptor CXCR4. This interaction is postulated to impair the function of tyrosine phosphatase, thereby altering the phosphorylation of molecules responsible for T cell signaling, and in turn activating apoptosis [84]. Probably, CD45 alters the PI3K/Akt pathway leading to apoptosis at the later stages of CD3/TCR activation [84]. Moreover, shed Gp120 upregulates FasL increasing the propensity to apoptosis [84]. Shed gp120 has also been shown to induce ADDC in uninfected T cells and infected T cells. However, the infected cells are protected by Nef and Vpu proteins as earlier described. The underlying molecular mechanism involves the binding of shed gp120 to CD4 of uninfected cells leading to the formation of a complex recognized by antibodies. The antibody-gp120/CD4 complex is recognized by the Fc receptor of NK cells leading to ADCC [85].

## 7. Future Perspectives

Indeed, the apoptotic pathway plays a crucial role in the pathogenesis of HIV. As explained above, HIV proteins modulate the activation of apoptosis in uninfected cells via different mechanisms to induce cell death. Targeting the secretion and effect of these proteins on uninfected cells can go a long way to improve the prognosis of HIV diseases. As explained above, most of these proteins (Nef, Tat, and Vpu) are secreted in exosomes, while others are shed (gp120) or secreted as soluble proteins (for example, Tat). Blocking the secretion of these proteins and exosomes using intracytoplasmic and membrane transporter blockers can be exploited as potential therapeutic strategies against HIV infection. Targeting and blocking the effects of these proteins in exosomes can be utilized as potential therapeutic strategies against HIV-induced bystander damage [16]. In addition, since gp120 binds to CD4 in uninfected cells to trigger apoptosis, the use of decoy CD4 molecules targeting gp120 can prevent bystander CD4+ T cell death [85]. Other mechanisms that can be exploited include the use of TRAILshorts which are capable of occupying TRAIL-R1/TRAIL-R2 without inducing cell cytotoxicity or apoptosis [86]. Nonetheless, more studies are required to understand clearly how these exosomes and proteins are secreted and transported to uninfected cells.

## Data Availability

All data cited or used in this review are included within the article.

## Abbreviations

HIV: Human immunodeficiency virus
TRECs: T cell receptor excision circles
TNFRSF: Tumor necrosis factor receptor subfamily
TRAIL: TNF-related apoptosis-inducing ligand
AIDS: Acquired immunodeficiency syndrome
TRAF2: TNF receptor-associated factor 2
RIPK1: Receptor-interacting serine/threonine protein kinase 1
TACE: TNF-*α*-converting enzyme
FADD: Fas-associated death domain
DISC: Death-inducing signaling complex
ICAD: Inhibitor of caspase-activated DNAse
ASK-1: Apoptosis-signaling regulatory kinase 1.
